# Morphological and Molecular Characterization of *Filenchus multistriatus* n. sp. (Tylenchomorpha: Tylenchidae) and Data on a Known Species of the Genus from Bushehr Province, Southern Iran

**DOI:** 10.2478/jofnem-2023-0008

**Published:** 2023-05-17

**Authors:** Somayeh Monemi, Mohammad Reza Atighi, Joaquín Abolafia, Pablo Castillo, Majid Pedram

**Affiliations:** 1 Department of Plant Pathology, Faculty of Agriculture, Tarbiat Modares University, Tehran, Iran; 2 Departamento de Biología Animal, Biología Vegetal y Ecología, Universidad de Jaén, Campus Las Lagunillas, s/n, 23071, Jaén, Spain; 3 Instituto de Agricultura Sostenible (IAS), Consejo Superior de Investigaciones Científicas (CSIC), Avenida Menéndez Pidal s/n, 14004, Córdoba, Spain

**Keywords:** *Filenchus sandneri*, LSU rDNA D2-D3, New species, SSU rDNA, Taxonomy, Tylenchinae

## Abstract

During a nematological survey in southern Iran, a population belonging to the family Tylenchidae was recovered from a tomato field in Bushehr province. The recovered population belongs to the genus *Filenchus,* was described and illustrated herein as *F. multistriatus* n. sp. It is mainly characterized by having a wide and low annulated lip region continuous with adjacent body; amphidial openings confined to the labial plate; four lines in lateral fields forming three bands, with the two outer bands broken by transverse, and the inner one broken by both transverse and longitudinal lines; and median bulb oval with visible valve and elongate-conoid tail uniformly and gradually narrowing toward the distal region, ending in a widely rounded tip. Its morphological and morphometric differences with three closely similar species were discussed. The phylogenetic relationships of the new species with other relevant genera and species were reconstructed using partial sequences of small, and large subunit ribosomal DNA (SSU and LSU rDNA) sequences. Morphometric and morphological data were also provided for an Iranian population of *F. sandneri* recovered from Bushehr province. Both populations were characterised using SEM data.

The subfamily Tylenchinae Örley, 1880 in the family Tylenchidae Örley, 1880 includes 15 genera according to Geraert (2008) and 11 genera according to Siddiqi (2000). *Labrys* Qing and Bert, 2018 is the last genus added to the subfamily. By excluding *Ottolenchus* spp. (Qing and Bert, 2017), the genus *Filenchus* Andrássy, 1954 currently contains 74 species (Geraert, 2008; Bert et al., 2010; Mortazavi et al., 2021), most of which were described based on traditional taxonomy approaches.

During a faunistic study on nematodes associated with fruit trees and vegetables in southern Iran, a Tylenchidae population having characteristics of *Filenchus* was found in a tomato field. The morphological and morphometric studies showed it has unique features and represents an unknown species. Hence, the present study aims to describe the new species using an integrative approach. Furthermore, additional data on an Iranian population of the species *Filenchus sandneri* Wasilewska, 1965 were included in this study.

## Materials and Methods

### Soil sampling, nematode extraction and morphological study

A total of 40 soil samples were collected from different gardens and fields and natural regions of the east Bushehr province, in southern Iran, in 2021. The nematodes were extracted from soil using the tray method (Whitehead and Hemming, 1965) and the nematodes of interest were handpicked under a Nikon SMZ1000 dissecting microscope. The specimens were heat-killed by adding hot 4% formaldehyde solution and then, transferred to anhydrous glycerine according to De Grisse (1969) and mounted on permanent slides. Measurements and drawings were performed using a drawing tube attached to a Nikon E600 light microscope and were digitally drawn using CorelDraw software version 2020. Light microphotographs were taken with a Nikon Eclipse 80i microscope (Nikon, Tokio, Japan) powered with differential interference contrast (DIC) and equipped with a Euromex sCEMX-6 digital camera (Euromex Microscopen BV, Arnhem, The Netherlands) (Abolafia, 2023). Indices and ratios were calculated according to Siddiqi (2000).

### Scanning electron microscopy (SEM)

Three females of each species preserved in glycerin were selected for SEM observations. The nematodes were hydrated in distilled water, dehydrated in a graded ethanol-acetone series, critical point dried, coated with gold, and observed with a Zeiss Merlin scanning electron microscope (5 kV) (Zeiss, Oberkochen, Germany) (Abolafia, 2015).

### DNA extraction, PCR and sequencing

DNA was extracted from three individual female specimens of the new species separately. Each specimen was squashed in 15 μl Tris EDTA buffer (10 mM Tris-Cl, 0.5 mM EDTA; pH 9.0) after examination on a temporary slide. The DNA samples were prepared and stored at -20°C until used as PCR templates. The SSU rDNA was amplified using forward primer 1096F (5′-GGTAATTCTGGAGCTAATAC-3′) and reverse primer 1912R (5′-TTTACGGTCAGAACTAG GG-3′); and forward primer 1813F (5′-CTGCGTG AGAGGTGAAAT-3′) and reverse primer 2426R (5′-GC TACCTTGTTACGACTTTT-3′) (Holterman et al., 2006). Primers for LSU rDNA D2-D3 amplification were forward primer D2Tyl (5′-GAGAGAGTTAAA NAGBACGTG-3′) (Oliveira et al., 2013) and reverse primer 1006R (5′-GTTCGATTAGTCTTTCG CCCCT-3′) (Holterman et al., 2008). The 30 μl PCR mixture contained: 15 μl *Taq* DNA Polymerase 2x Master Mix RED, 2mM MgCl2 (Ampliqon-Denmark), 8 μl distilled water, 1 μl of each primer, and 5 μl of DNA template. The thermocycling program for amplification of both loci was as follows: denaturation at 95°C for 4 min, followed by 35 cycles of denaturation at 94°C for 30 sec, annealing at 52°C for 40 sec, and extension at 72°C for 80 sec. A final extension was performed at 72°C for 10 min. The PCR products were sequenced with an ABI 3730XL sequencer (Bioneer Corporation, South Korea) in both directions using the same primers applied for amplification of that segment. The newly generated sequences were deposited into the GenBank database under the accession numbers OM914650 for SSU, and OM914648 and OM914649 for LSU rDNA D2-D3 of the new species.

### Phylogenetic analyses

The quality of the newly obtained sequences was manually checked using Chromas software (http://technelysium.com.au/wp/chromas), edited and assembled. Each of the SSU and LSU sequences was compared with other available sequences in the GenBank database using the basic local alignment search tool (BLAST) homology search program. Several relevant sequences of representatives of the family Tylenchidae were selected for both SSU and LSU phylogenies. Representatives of Aphelenchoidea Fuchs, 1937, including *Aphelenchoides fragariae* (Ritzema Bos, 1890), Christie, 1932 (AY284645), and *Bursaphelenchus mucronatus* Mamiya & Enda, 1979 (AY284648) for SSU, as well as *B. acalolepta* Kanzaki, Ekino, Maehara, Aikawa & Giblin-Davis 2020 (AB650013) and *B. luxuriosae* Kanzaki & Futai, 2003 (AB299228) for LSU, were used as outgroups. Both datasets were aligned using MUSCLE (Edgar, 2004) and manually edited using MEGA6 (Tamura et al., 2013). The best-fitting substitution model for both datasets was selected using PAUP*/MrModeltest2 (Nylander, 2004). The Akaike supported model, a general time reversible model, including among site rate heterogeneity and estimates of invariant sites (GTR + G + I) was selected and used in both phylogenies. Bayesian analysis was performed using MrBayes 3.1.2 (Ronquist and Huelsenbeck, 2003) with running the chains for 5×10^6^ generations for both datasets. After discarding burn-in samples, the remaining samples were retained for further analyses. The Markov chain Monte Carlo (MCMC) method within a Bayesian framework was used to estimate the posterior probabilities of the phylogenetic trees (Larget and Simon, 1999) using the 50% majority rule. The convergence of model parameters and topology were assessed based on average standard deviation of split frequencies and potential scale reduction factor values. Adequacy of the posterior sample size was evaluated using autocorrelation statistics as implemented in Tracer v.1.6 (Rambaut and Drummond, 2009). The output files of the trees were visualized using Dendroscope v3.2.8 (Huson and Scornavacca, 2012) and digitally drawn in CorelDRAW software version 2020.

## Results

### Systematics

#### Filenchus multistriatus n. sp.

**Figure 1:**
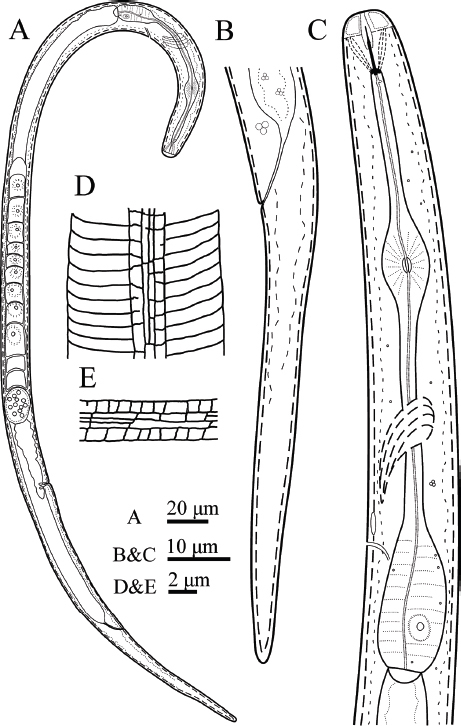
Line drawings of *Filenchus multistriatus* n. sp. (Female). (A) Entire body. (B) Tail. (C) Pharynx. (D) Lateral view of lateral field. (E) Lateral field.

**Figure 2:**
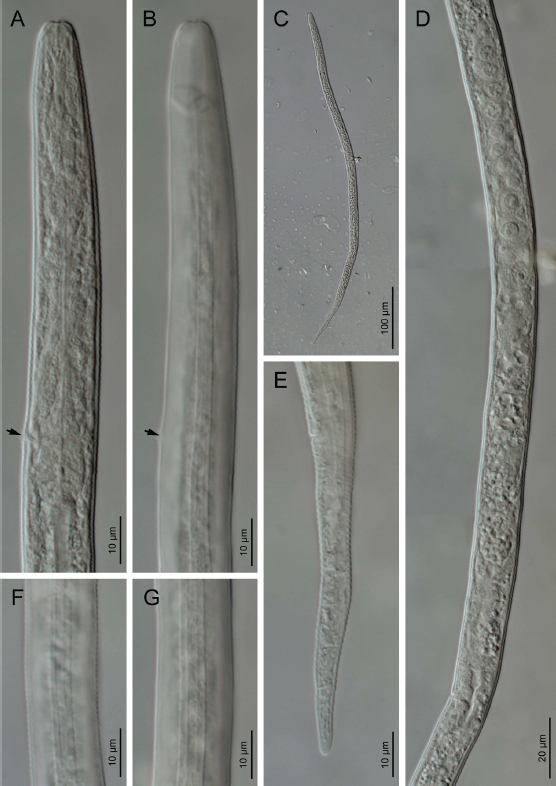
Light microphotographs of *Filenchus multistriatus* n. sp. (Female). (A&B) Anterior body region (arrows pointing to the excretory pore). (C) Entire body. (D) Reproductive system. (E) Tail. (F&G) Lateral field in two paratypes in lateral view.

**Figure 3:**
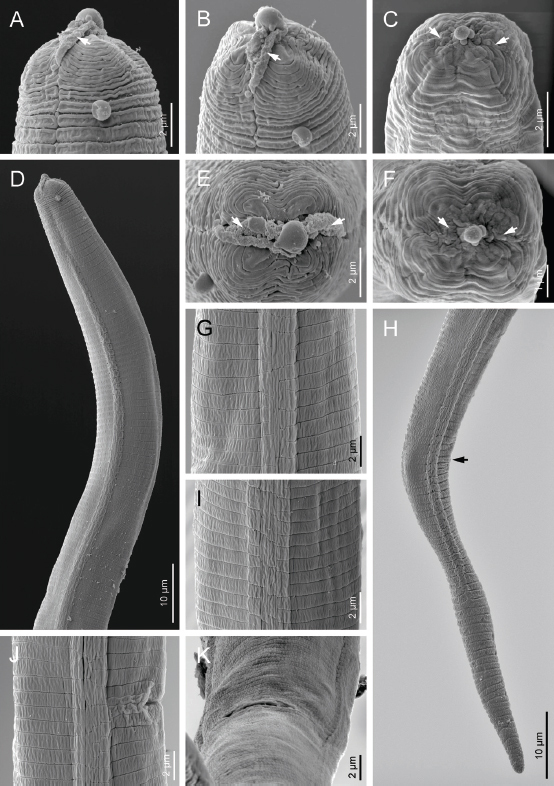
Scanning electron microphotographs of *Filenchus multistriatus* n. sp. (Female). (A-C) Lip region in ventral and subfrontal views, respectively (arrows pointing to the amphidial openings). (D) Anterior body region. (E&F) Lip region in frontal view (arrows pointing to the amphidial openings). (G, I, J) Lateral field. (H) Tail (arrow pointing to the anus). (K) Vulva.

(Figs. 1-3; Table 1).

**Table 1.** Morphometrics of *Filenchus multistriatus* n. sp. and *F. sandneri* Wasilewska, 1965 from Bushehr province, Iran. All measurements are in μm and in the form: mean ± SD (range).

	*F. multistriatus* n. sp.		*F. sandneri*
Character	Holotype	Paratypes	Bushehr population
Sex	Female	Female	Female
n	1	11	9
L	567	537.3±33.0 (500-590)	400.1±48.0 (331-454)
L′	497	464.4±33.1 (423-515)	352.8±46.0 (288-406)
a	40.5	35.0±3.5 (29.3-40.5)	31.1±4.0 (25.5-37.6)
b	5.3	5.5±0.3 (5.0-6.0)	5.1±0.5 (4.2-5.9)
c	8.1	7.4±0.5 (6.4-8.4)	8.4±0.8 (7.3-9.5)
c′	7.0	7.2±0.9 (5.8-8.3)	5.7±0.3 (5.3-6.5)
V	70.5	69.6±3.0 (63.2-73.8)	73.1±2.5 (68.1-75.8)
V′	80.5	81.5±1.9 (77.8-84.9)	83.7±1.8 (81.4-86.2)
Lip region height	1.8	1.8±0.1 (1.7-1.9)	2.1±0.3 (1.5-2.4)
Lip region width	0.7	6.8±0.3 (6-7)	5.0±0.2 (4.5-5.3)
Stylet	6.6	7.1±0.4 (6.5-7.6)	8.0±0.5 (7.5-8.6)
Conus	2.1	2.5±0.4 (2-3)	2.8±0.2 (2.6-3.0)
m	31.8	35.5±3.5 (30.8-39.5)	35.4±1.8 (32.5-38.5)
DGO	0.7	0.7±0.1 (0.5-0.8)	2.7±0.2 (2.5-3.0)
Excretory pore	81	75.4±5.5 (67-81)	57.7±5.5 (50-64)
Median bulb	47	38.2±4.4 (35-47)	- -
MB	43.9	38.7±3.9 (34.7-48.5)	35.8±2.8 (30.5-41.3)
Pharynx	107	98.6±3.8 (94-107)	78.1±5.9 (71-90)
Body width (BW)	14	15.5±2.0 (14-20)	12.9±0.9 (12-14)
Anterior end to vulva	400	374±35 (316-427)	292.8±40.3 (236-343)
Vulva - anus	97	85±11 (67-105)	57.0±8.4 (40-70)
Anal body width	10	10.3±1.0 (9-12)	8.3±0.5 (8-9)
PUS	12	12.2±0.7 (11-13)	8.3±0.4 (7.8-8.8)
PUS/BW	0.9	0.8±0.1 (0.6-0.9)	8.3±0.4 (7.8-8.8)
Tail	70	72.9±3.1 (70-78)	47.3±3.1 (43-52)
Tail/V-A	0.7	0.9±0.1 (0.7-1.2)	0.8±0.1 (0.6-1.2)

### Description

#### Female

Body straight to irregularly curved. Cuticle with fine annuli under LM. The body annuli sometimes broken by irregular transverse lines in SEM images. Lateral fields with four longitudinal incisures or three bands in LM (Figs. 2B, F, G). SEM shows the two outer bands broken by transverse, and inner band broken by both transverse and longitudinal lines (Figs. 3G, I, J). Lip region continuous with adjacent body, low and wide. Lip region annulated, amphidial openings confined to the labial plate in the SEM image. Stylet weak and fine, the conus about one-third of the total stylet, knobs small, posteriorly directed. Orifice of dorsal gland of pharynx (DGO) just behind stylet knobs. Pharynx tylenchoid, the procorpus slender, joining an oval metacorpus with valvular apparatus, isthmus narrower and longer than procorpus, and basal bulb saccate. Nerve ring encircling isthmus at about the middle. Excretory pore at the level of the anterior part of the basal bulb. Hemizonid slightly anterior to excretory pore. Intestine simple, rectum and anus functional. Reproductive system monodelphic-prodelphic, composed of an outstretched ovary with the oocytes in a single row, tubular oviduct at its distal part, rounded to slightly oval offset spermatheca filled with spheroid sperm, crustaformeria apparently quadricolumellate and uterus with visible lumen and thin walls, vagina with thin walls, perpendicular to the body axis and post-vulval uterine sac (PUS) short, 0.6-0.9 times of vulval body diameter. Tail elongate conical, straight, ending to a widely rounded tip.

#### Male

Not found.

### Type habitat and locality

The new species was recovered from a soil sample collected from a tomato farm in Serajabad village (south of Dashtestan), Bushehr province, southern Iran, in 23 December 2020. GPS coordinates: 29°29.609′N, 050°56.803′E.

### Type material

Holotype female and eleven paratype females were deposited into the WaNeCo nematode collection (http://www.waneco.eu/), The Netherlands.

### LSID for this publication

B706568E-35F1-4719-A03C-5520FD2FDF7C

### Etymology

The specific epithet refers to the numerous short longitudinal and transversal striations or incisures appearing at the inner band of the lateral fields.

### Diagnosis and relationships

The new species is mainly characterized by having a wide and low lip region continuous with adjacent body, annulated in SEM observations, amphidial apertures as short slits confined to the labial plate under SEM, four lines in lateral fields forming three bands, the two outer bands broken by transverse, and inner one broken by both transverse and longitudinal lines and an elongate conoid tail with wide rounded tip. The new species was morphologically compared with three species *Filenchus crassus* Siddiqui & Khan, 1983, *F. hamatus* and *F. sandneri* as follow (morphological and morphometric data of *F. hamatus* after Brzeski (1997); and those of the two latter species according to their original descriptions):

From *F. crassus* by having a longer body (500-590 *vs* 350-400 μm) and longer pharynx (94-107 *vs* 74.5 μm in holotype).

From *F. hamatus* by having a wider and lower lip region (*vs* high, trapezoid) and straight *vs* ventrally bent (hooked) tail.

From *F. sandneri* by having a wider and lower lip region (*vs* high), longer body (500-590 *vs* 370-470 μm), smaller V (63.2-73.8 *vs* 73-75 μm) and straight tail (*vs* ventrally bent).

### Bushehr population of *Filenchus sandneri*

**Figure 4:**
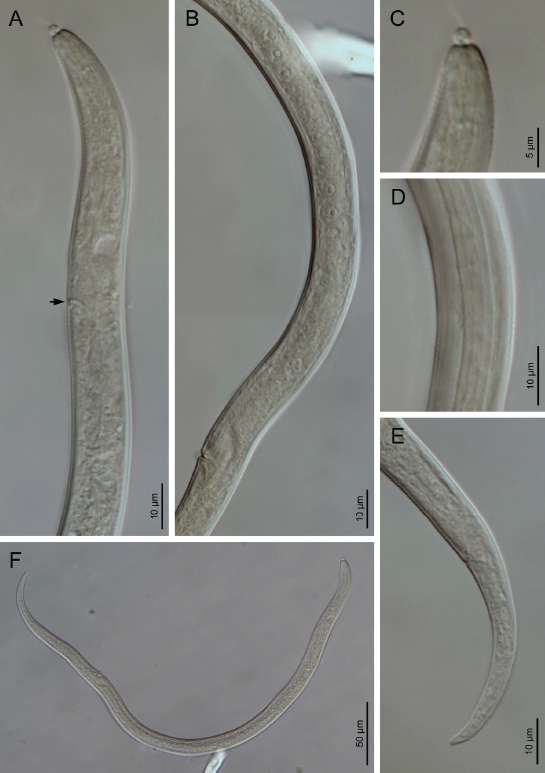
Light microphotographs of Iranian population of *F. sandneri* Wasilewska, 1965 from Bushehr province (Female). (A) Anterior body region (arrows pointing to the excretory pore). (B) Reproductive system. (C) Lip region and stylet. (D) Lateral field. (E) Tail. (F) Entire body.

**Figure 5:**
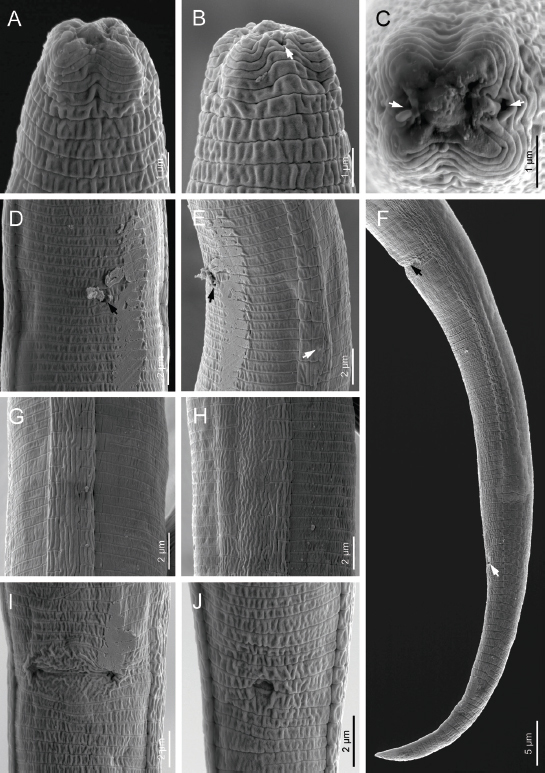
Scanning electron microphotographs of Iranian population of *F. sandneri* Wasilewska, 1965 from Bushehr province (Female). (A-C) Lip region in ventral, subfrontal and frontal views, respectively (arrows pointing to the amphidial openings). (D) Excretory pore. (E) Lateral field. (F) Tail (upper and lower arrows pointing to the vulva and anus, respectively). (G,H) Lateral fields. (I) Vulva. (J) Anus.

(Figs. 4,5; Table 1).

### Remarks

The Dashtestan population of this species is in full morphological and morphometric agreement with the type population. It has been reported several times from Iran (Karegar, 2018). The range of morphometric data of this species has been expanded after several reports (Geraert, 2008). According to our SEM data (Fig. 5), the lip region is annulated and amphidial openings are small pores close to the labial plate. In the previous SEM study of species (Karegar and Geraert 1998), the amphidial openings were not seen. The identity of the population(s) reported as *Filenchus* cf. *sandneri* in the latter study, however, needs further validation, as there are remarkable differences between their morphometric data compared with those of the type population.

### Molecular phylogenetic status

Sequencing of SSU and LSU rDNA D2-D3 fragments of the new species yielded a single 1592 nucleotide long SSU (accession number OM914650); and two 605 nucleotide long LSU sequences (accession numbers OM914648 and OM914649).

**Figure 6:**
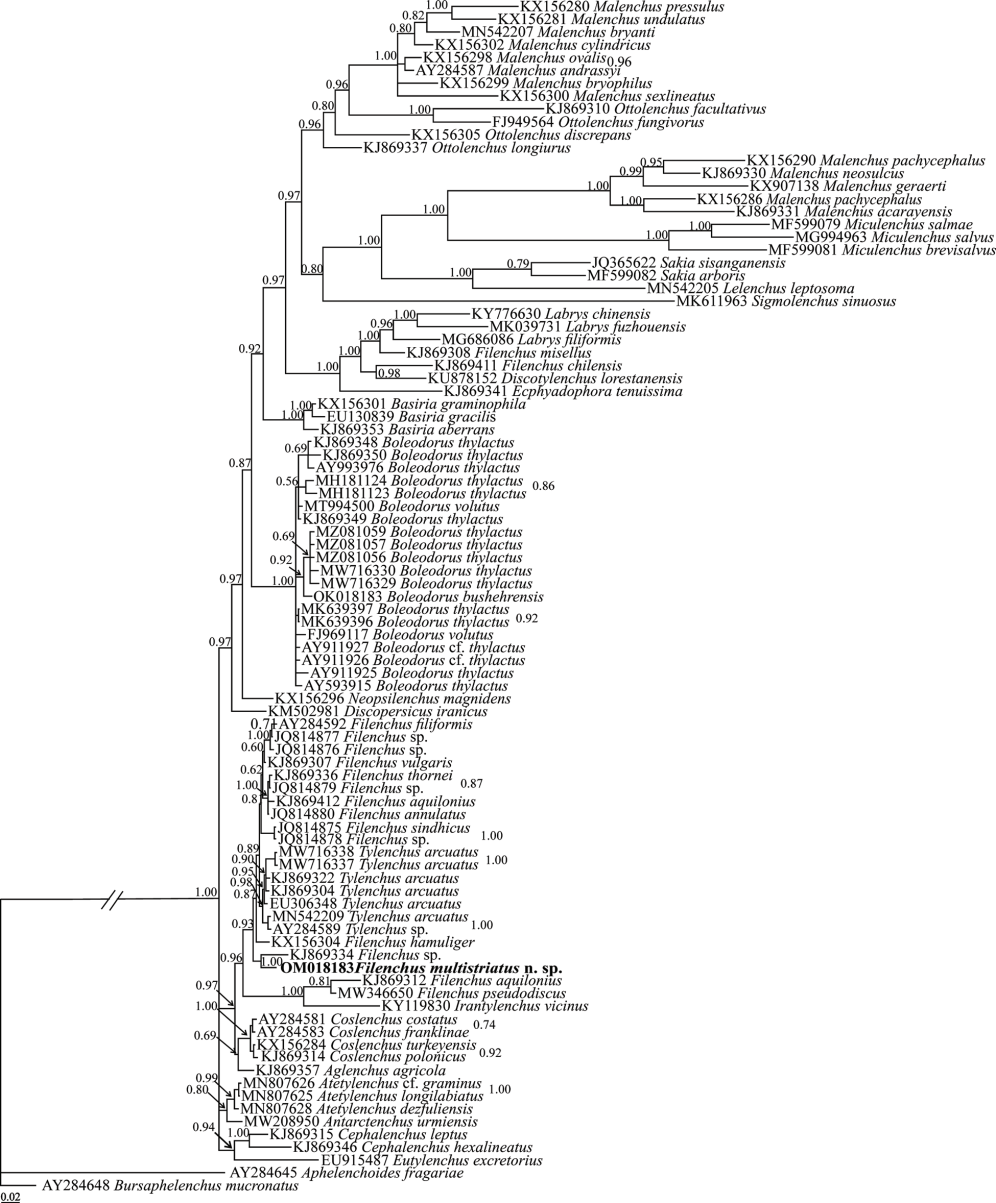
Bayesian 50% majority rule consensus tree inferred from SSU rDNA of *Filenchus multistriatus* n. sp. under the GTR + G + I model (-ln L = 20913.9590; freq A = 0.2301; freqC = 0.2419; freqG = 0.3006; freq T = 0.2273; R(a) = 0.8974; R(b) = 2.1547; R(c) = 0.9066; R(d) = 1.0666; R(e) = 4.2155; R(f) = 1.0000; Pinva = 0.2800; Shape = 0.4692). Bayesian posterior probability values are given for appropriate clades. The newly generated sequence of the new species is in bold font.

The BLAST search using the newly generated SSU sequence, revealed it has a 96.87-97.00% identity with six sequences assigned to *Tylenchus arcuatus* Siddiqi, 1963 (MW716338, MW716337, EU306348, KJ869322, KJ869304, MN542209). Its identity with the sequence assigned to *Filenchus vulgaris* Brzeski, 1963 (KJ869307) was 96.87%. Its identity with all other SSU sequences was less than 96.87%. A number of 93 SSU sequences (including newly generated sequence of the new species and two aphelenchoidid sequences as outgroups) were included in SSU phylogeny (for accession numbers, see the SSU tree). The SSU phylogenetic tree in Fig. 6 includes sequences of *Filenchus* which have occupied different placements, corroborating its non-monophyletic condition according with this marker. The SSU sequence of the new species has formed a maximally supported clade with an SSU sequence (KJ869334) assigned to *Filenchus* sp. This clade is in sister relation with a clade including several sequences of *Filenchus* spp. (AY284592, JQ814877, KJ869307, JQ814879, KJ869336, JQ814880, JQ814876, KX156304, JQ814875, JQ814878, KJ869412) and the sequences assigned to *Tylenchus arcuatus* (EU306348, MW716338, MW716337, KJ869322, KJ869304, MN542209) and *Tylenchus* sp. (AY284589).

**Figure 7:**
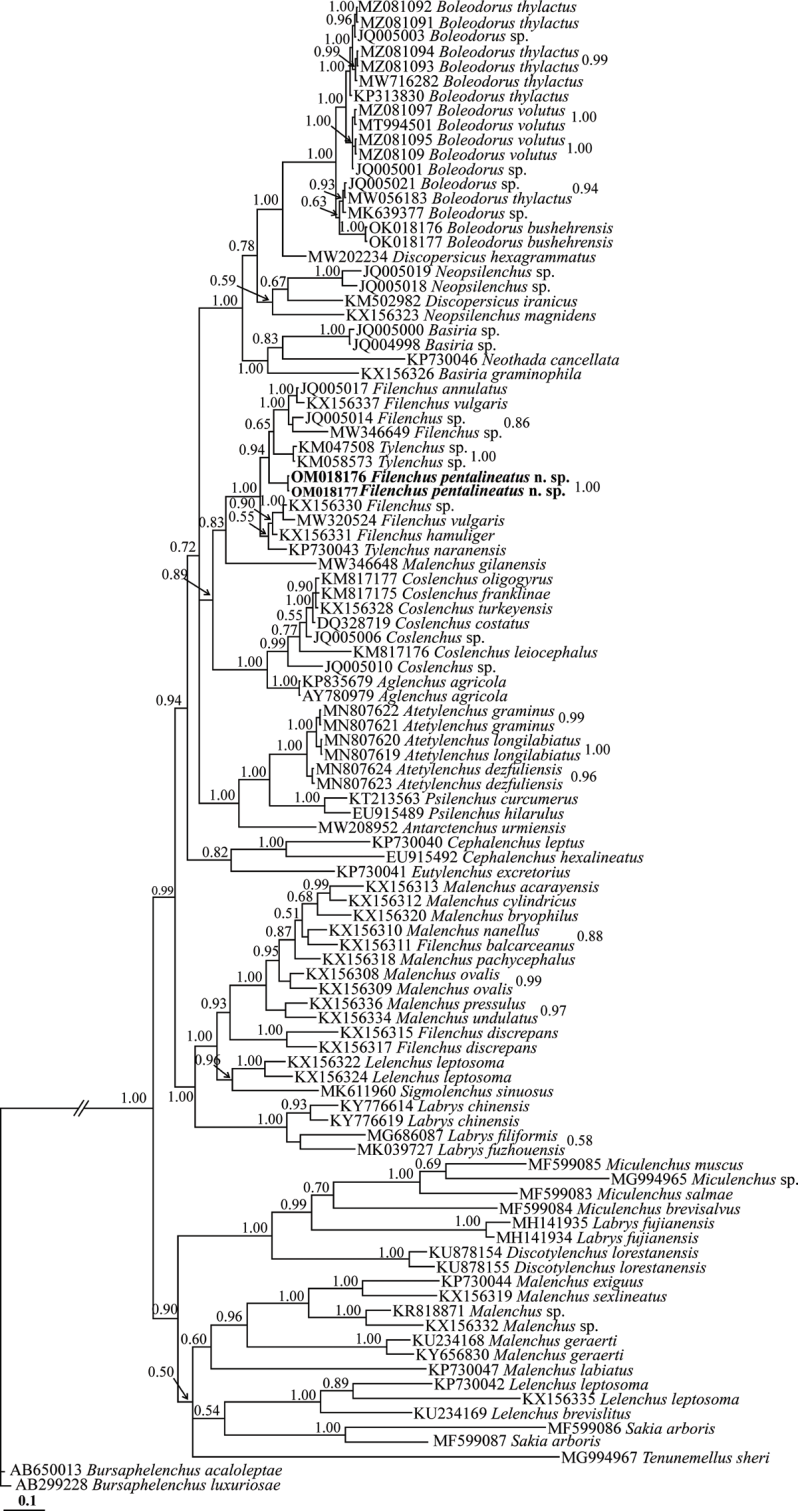
Bayesian 50% majority rule consensus tree inferred from LSU rDNA D2-D3 sequences of *Filenchus multistriatus* n. sp. under the GTR+G+I model (-ln L = 22199.4473; freq A = 0.1507; freqC = 0.2476; freqG = 0.3242; freq T = 0.2775; R(a) = 0.8948; R(b) = 0.8948; R(c) = 1.0152; R(d) = 0.7990; R(e) = 3.4497; R(f) = 1.0000; Pinva = 0.1528; Shape = 0.7240). Bayesian posterior probability values are given for appropriate clades. The newly generated sequences of the new species are in bold font.

The BLAST search using the LSU sequences of the new species revealed that their identity with all currently available LSU sequences of Tylenchidae is less than 91% (the highest identity was 90.76 %, belonging to *Filenchus* sp. (KX156330)). A number of 102 sequences including the newly generated sequences and two sequences of outgroup taxa were used for LSU phylogeny. In this tree (Fig. 7), several species of *Filenchus* have occupied different placements. The two LSU sequences of the new species have formed a clade with sequences assigned to *Filenchus* spp. (JQ005017, KX156337, JQ005014, MW346649) and *Tylenchus* sp. (KM047508, KM058573).

## Discussion

In the present study, one new Tylenchinae species was recovered from southern Iran. It was assigned to the genus *Filenchus* by having a stylet conus about one third of the total length and small amphidial openings confined to labial plate under SEM, being confirmed by SSU and LSU phylogenies. The new species has a low flat lip region, which has already been reported for some other Tylenchidae spp. (Geraert, 2009; Monemi et al., 2022).

Most Tylenchidae species have been established based solely on traditional criteria, and several reports have extended their morphological data ranges (Geraert, 2008). Furthermore, the interpretation of morphological and morphometric differences as “intraspecies variations” may yield to incorrect assignments of given populations to species. In a study by Monemi et al. (2022), it has been shown that several sequences assigned to *Boleodorus thylactus* Thorne, 1941 have occupied different placements in corresponding trees, and as the conclusion, the original descriptions of the Tylenchidae species or data given in redescriptions of the species based on type specimens, are reliable resources in taxonomic studies of this group of nematodes. In accordance with some recent phylogenies using ribosomal markers (e.g. Panahandeh et al., 2018; Qing and Bert, 2018), the two currently resolved phylogenies using both SSU and LSU ribosomal markers show the genus *Filenchus* is polyphyletic.

## References

[j_jofnem-2023-0008_ref_001] Abolafia J. (2015). A low-cost technique to manufacture a container to process meiofauna for scanning electron microscopy. Microscopy Research and Technique.

[j_jofnem-2023-0008_ref_002] Abolafia J. (2023). Metodología para la realización de análisis morfológicos y morfométricos en nematodos de muestras de suelos de cuevas y otros hábitats. Monografías Bioespeleológicas.

[j_jofnem-2023-0008_ref_003] Andrássy I. (1954). Revision der Gattung *Tylenchus* Bastian, 1865 (Tylenchidae, Nematoda). Acta Zoologica Hungaricae.

[j_jofnem-2023-0008_ref_004] Bert W., Okada H., Tavernier I., Borgonie G., Houthoofd W. (2010). Morphological, morphometric and molecular characterisation of *Filenchus fungivorus* n. sp., a fungivorous nematode from Japan in a most likely polyphyletic genus (Nematoda: Tylenchina). Nematology.

[j_jofnem-2023-0008_ref_005] Brzeski M. W. (1963). On the taxonomic status of *Tylenchus filiformis* Butschli, 1873, and the description of *T. vulgaris* sp. n. (Nematoda: Tylenchidae). Bulletin de l’Academie Polonaise des Sciences.

[j_jofnem-2023-0008_ref_006] Christie J. R. (1932). Recent observations on the strawberry dwarf nematode in Massachusetts. Plant Disease Reporter.

[j_jofnem-2023-0008_ref_007] De Grisse A. T. (1969). Redescription ou modifications de quelques technique utilisées dan l’etude des nematodes phytoparasitaires. Mededelingen Rijksfaculteit Landbouwwetenschappen Gent.

[j_jofnem-2023-0008_ref_008] Edgar R. C. (2004). MUSCLE: multiple sequence alignment with high accuracy and high throughput. Nucleic Acids Research.

[j_jofnem-2023-0008_ref_009] Eroshenko A. S. (1971). [Nematoda *Tylenchus* Bastian, 1865] (in Russian). Parazity Zhivothykh i rosteniy Dal’nego Vostoka.

[j_jofnem-2023-0008_ref_010] Fuchs A. G. (1937). Neue parasitische undhalbparasitische Nematoden bei Borkenkäfern und einige andere Nematoden. I. Teil. Zoologische Jahrbücher, Abteilung für Systematik, Ökologie und Geographie der Tiere.

[j_jofnem-2023-0008_ref_011] Geraert E. (2008). The Tylenchidae of the world: identification of the family Tylenchidae (Nematoda). Gent.

[j_jofnem-2023-0008_ref_012] Holterman M., Rybarczyk K., van den Elsen S., van Megen H., Mooyman P., Peña Santiago R., Bongers T., Bakker J., Helder J. (2008). A ribosomal DNA-based framework for the detection and quantification of stress-sensitive nematode families in terrestrial habitats. Molecular Ecology Resources.

[j_jofnem-2023-0008_ref_013] Holterman M., Vander Wurff A., Vanden Elsen S., Van Megen H., Bongers T., Holovachov O., Bakker J., Helder J. (2006). Phylum-wide analysis of SSU rDNA reveals deep phylogenetic relationships among nematodes and accelerated evolution toward crown clades. Molecular Biology and Evolution.

[j_jofnem-2023-0008_ref_014] Huson D. H., Scornavacca C. (2012). Dendroscope 3: an interactive tool for rooted phylogenetic trees and networks. Systematic Biology.

[j_jofnem-2023-0008_ref_015] Kanzaki N., Futai K. (2003). Description and phylogeny of *Bursaphelenchus luxuriosae* n. sp. (Nematoda: Aphelenchoididae) isolated from Acalolepta luxuriosa (Coleoptera: Cerambycidae). Nematology.

[j_jofnem-2023-0008_ref_016] Kanzaki N., Ekino T., Maehara N., Aikawa T., Giblin-Davis R.M. (2020). *Bursaphelenchus acaloleptae* n. sp. sharing tree and beetle carrier hosts with *B. luxuriosae* Kanzaki & Futai, 2003 in Japan. Nematology.

[j_jofnem-2023-0008_ref_017] Karegar A., Ghaderi R., Kashi L., Karegar A. (2018). Plant-parasitic nematodes in Iran.

[j_jofnem-2023-0008_ref_018] Larget B., Simon D. L. (1999). Markov chain Monte Carlo algorithms for the Bayesian analysis of phylogenetic trees. Molecular Biology and Evolution.

[j_jofnem-2023-0008_ref_019] Mamiya Y., Enda N. (1979). *Bursaphelenchus mucronatus* n. sp. (Nematoda: Aphelenchoididae) from pinewood and its biology and pathogenicity to pine trees. Nematologica.

[j_jofnem-2023-0008_ref_020] Mortazavi P., Heydari F., Abolafia J., Castillo P., Pedram M. (2021). Morphological and molecular characterization of *Filenchus pseudodiscus* n. sp. from east Golestan province, north Iran; with an updated phylogeny of *Malenchus* Andrássy, 1968 (Nematoda: Tylenchidae). Journal of Nematology.

[j_jofnem-2023-0008_ref_021] Monemi S., Atighi M., Abolafia J., Pourjam E., Pedram M. (2022). Description of *Boleodorus bushehrensis* n.sp. (Rhabditida: Tylenchidae) from southern Iran, and observation as a commonly known species. Journal of Nematology.

[j_jofnem-2023-0008_ref_022] Nylander J. A. (2004). MrModeltest v2. Evolutionary Biology Centre.

[j_jofnem-2023-0008_ref_023] Oliveira R. D. L., Santin Â. M., Seni D. J., Dietrich A., Salazar L. A., Subbotin S. A., Mundo-Ocampo M., Goldenberg R., Barreto R. W. (2013). *Ditylenchus gallaeformans* n. sp. (Tylenchida: Anguinidae) a neotropical nematode with bio-control potential against weedy Melastomataceae. Nematology.

[j_jofnem-2023-0008_ref_024] Örley L. (1880). Monograph of the Anguillulids. Természeti Füzetek.

[j_jofnem-2023-0008_ref_025] Panahandeh Y., Abolafia J., Pourjam E., Jahanshahi Afshar F., Giblin-Davis R. M., Pedram M. (2018). Morphological and molecular characterization of *Labrys filiformis* n. sp. (Rhabditida: Tylenchidae) from Iran. Journal of Nematology.

[j_jofnem-2023-0008_ref_026] Qing X., Bert W. (2017). Redefinition of genus *Malenchus* Andrássy, 1968 (Tylenchomorpha: Tylenchidae) with additional data on ecology. Journal of Nematology.

[j_jofnem-2023-0008_ref_027] Qing X., Bert W. (2018). 3D printing in zoological systematics: integrative taxonomy of *Labrys chinensis* gen. nov., sp. nov. (Nematoda: Tylenchomorpha). Journal of Zoological Systematics and Evolutionary Research.

[j_jofnem-2023-0008_ref_028] Rambaut A., Drummond A. J. (2009). Tracer version 1.5 (computer program).

[j_jofnem-2023-0008_ref_029] Ronquist F., Huelsenbeck J. P. (2003). MrBAYES 3: Bayesian phylogenetic inference under mixed models. Bioinformatics.

[j_jofnem-2023-0008_ref_030] Siddiqi M. R. (1963). Four new species of the genus *Tylenchus* Bastian, 1865 (Nematoda) from North India. Zeitschrift für Parasitenkunde.

[j_jofnem-2023-0008_ref_031] Siddiqui A. U., Khan E. (1983). Taxonomic studies on Tylenchidae (Nematoda) of India. V: Three new species of the genus *Lelenchus* (Andrássy, 1954) Meyl, 1960 from India. Indian Journal of Nematology.

[j_jofnem-2023-0008_ref_032] Siddiqi M.R. (1986). Tylenchida: Parasites of Plants and Insects.

[j_jofnem-2023-0008_ref_033] Siddiqi M. R. (2000). Tylenchida: Parasites of Plants and Insects.

[j_jofnem-2023-0008_ref_034] Tamura K., Stecher G., Peterson D., Filipski A., Kumar S. (2013). MEGA6: molecular evolutionary genetics analysis version 6.0. Molecular Biology and Evolution.

[j_jofnem-2023-0008_ref_035] Thorne G. (1941). Some nematodes of the family Tylenchidae which do not possess a valvular median esophageal bulb. Great Basin Naturalist.

[j_jofnem-2023-0008_ref_036] Thorne G., Malek R. B. (1968). Nematodes of the Northern Great Plains. Part 1. Tylenchida (Nemata: Secernentea). South Dakota Agricultural Experiment Station Technical Bulletin:.

[j_jofnem-2023-0008_ref_037] Whitehead A. G., Hemming J. R. (1965). A comparison of some quantitative methods of extracting small vermiform nematodes from soil. Annals of Applied Biology.

