# Erratum to: Morphological and Molecular Characterization of *Filenchus multistriatus* n. sp. (Tylenchomorpha: Tylenchidae) and Data on a Known Species of the Genus from Bushehr Province, Southern Iran

**DOI:** 10.2478/jofnem-2023-0046

**Published:** 2023-12-31

**Authors:** Somayeh Monemi, Mohammad Reza Atighi, Joaquín Abolafia, Pablo Castillo, Majid Pedram

**Affiliations:** Department of Plant Pathology, Faculty of Agriculture, Tarbiat Modares University, Tehran, Iran; Departamento de Biología Animal, Biología Vegetal y Ecología, Universidad de Jaén, Campus Las Lagunillas, s/n, 23071, Jaén, Spain; Instituto de Agricultura Sostenible (IAS), Consejo Superior de Investigaciones Científicas (CSIC), Avenida Menéndez Pidal s/n, 14004, Córdoba, Spain

In the SSU phylogenetic tree (Fig. 6), the accession number of the newly generated sequence should be OM914650.

In the LSU phylogenetic tree (Fig. 7), the name of the new species should be *Filenchus multistriatus* n. sp. and the accession numbers should be OM914648 and OM914649.

